# Rno_circ_0001004 Acts as a miR-709 Molecular Sponge to Regulate the Growth Hormone Synthesis and Cell Proliferation

**DOI:** 10.3390/ijms23031413

**Published:** 2022-01-26

**Authors:** Jiali Xiong, Haojie Zhang, Yuxuan Wang, Yunyun Cheng, Junyi Luo, Ting Chen, Qianyun Xi, Jiajie Sun, Yongliang Zhang

**Affiliations:** College of Animal Science, Guangdong Provincial Key Lab of Agro-Animal Genomics and Molecular Breeding, National Engineering Research Center for Breeding Swine Industry, South China Agricultural University, Guangzhou 510642, China; XJL00LJX@163.com (J.X.); zhanghj089@126.com (H.Z.); 13980686685@163.com (Y.W.); chengyy@jlu.edu.cn (Y.C.); luojunyi@scau.edu.cn (J.L.); allinchen@scau.edu.cn (T.C.); xqy0228@163.com (Q.X.)

**Keywords:** pituitary, rno_circ_0001004, miR-709, GH, proliferation

## Abstract

(1) Background: As a novel type of non-coding RNA with a stable closed-loop structure, circular RNA (circRNA) can interact with microRNA (miRNA) and influence the expression of miRNA target genes. However, circRNA involved in pituitary growth hormone (GH) regulation is poorly understood. Our previous study revealed protein kinase C alpha (*PRKCA*) as the target gene of miR-709. Currently, the expression and function of rno_circRNA_0001004 in the rat pituitary gland is not clarified; (2) Methods: In this study, both bioinformatics analysis and dual-luciferase report assays showed a target relationship between rno_circRNA_0001004 and miR-709. Furthermore, the rno_circRNA_0001004 overexpression vector and si-circ_0001004 were constructed and transfected into GH_3_ cells; (3) Results: We found that rno_circRNA_0001004 expression was positively correlated with the PRKCA gene and GH expression levels, while it was negatively correlated with miR-709. In addition, overexpression of rno-circ_0001004 also promoted proliferation and relieved the inhibition of miR-709 in GH_3_ cells; (4) Conclusions: Our findings show that rno_circ_0001004 acts as a novel sponge for miR-709 to regulate GH synthesis and cell proliferation, and are the first case of discovery of the regulatory role of circRNA_0001004 in pituitary GH.

## 1. Introduction

Growth hormone (GH) is a key hormone secreted from the anterior pituitary, and has received much attention as it regulates key physiological functions such as growth and development [1]. Studies have shown that microRNAs (miRNAs) can regulate the synthesis and secretion of GH [2,3,4]. MiRNAs are small single endogenous RNAs that regulate post-transcriptional silencing of target genes by binding to the 3′-untranslated region (UTR) or open reading frame (ORF) region of target mRNAs [5]. More and more evidence has demonstrated that miRNA functions broadly in development, physiology, and pathology by influencing cell proliferation, cell differentiation, cell migration, apoptosis, metabolism and signal transduction [6,7,8,9,10,11]. We have previously shown that the miR-709 is highly expressed in the pituitary and inhibits the GH synthesis and suppresses the viability of GH_3_ cells [12] by targeting Protein Kinase C alpha (*PKCA*). Protein Kinase C (PKC) is a class of phospholipid-dependent kinases that participate in regulation of protein secretion including GH and luteinizing hormone (LH) [13,14], as well as regulating cell proliferation [15].

Circular RNA (circRNA) is a special type of non-coding RNA (ncRNA) molecule that, unlike traditional linear RNA, forms covalently closed loop structures generated by pre-mRNA back splicing. CircRNAs are highly stable, abundant and conserved molecules with the characteristics of cell tissue specificity [16] and have received more attention due to their multiple regulation functions in animals and plants [17,18,19]. Similar to other regulatory ncRNAs, circRNAs play important roles in various biological processes, such as acting as a scaffold in the assembly of protein complexes [20,21,22], regulating alternative RNA splicing or transcription and RNA-protein interactions [23,24] and functioning as competing endogenous RNA (ceRNA) [25] or microRNA (miRNA) sponges [26,27,28,29,30,31]. However, reports of circRNAs involved in pituitary GH regulation have been very scarce up to the present [32,33]. 

The rno_circ_0001004 was firstly discovered in the rat anterior pituitary by using Illumina sequencing [34]. It is generated from exons 9 to 11 of the Wnk2 gene, with a length of 888 bp. However, the underlying regulatory role of rno_circ_0001004 in the pituitary remains unknown. Thus, in the present study, the role of rno_circ_0001004 in the regulation of GH and cell proliferation and the circRNA-miRNA-mRNA network were explored and identified.

## 2. Results

### 2.1. Characterization of Rno_circ_0001004 in GH_3_ Cells

**Figure 1:**
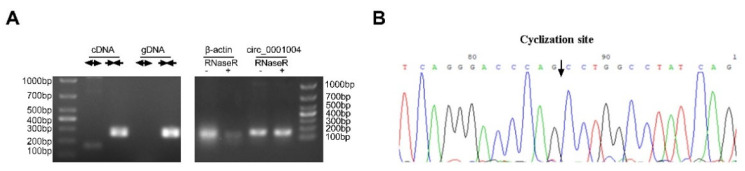
Characterization of rno_circ_0001004 in a GH_3_ cell. (**A**) PCR analysis for rno_circ_0001004 in the cDNA and gDNA of a GH_3_ cell.(left) Total RNA from GH_3_ cells with or without RNase R treatment was subjected to RT-PC.(right) (**B**) The back-splice junction of rno_circ_0001004 was identified by Sanger sequencing.

To characterize rno_circ_0001004 in GH_3_ cells, we firstly detected the expression of rno_circ_0001004. Convergent and divergent primers were designed to amplify the linear or back-splicing products and total RNA from GH_3_ cells with or without RNase R treatment was subjected to RT-PCR. As expected, endogenous circ_0001004, but not pre-mRNA, was resistant to RNase R digestion (Figure 1A). Sanger sequencing validated the back-spliced junction of rno_circ_0001004 (Figure 1B).

### 2.2. Rno_circ_0001004 Antagonizes miR-709-Mediated Repression of GH Synthesis and Secretion

**Figure 2:**
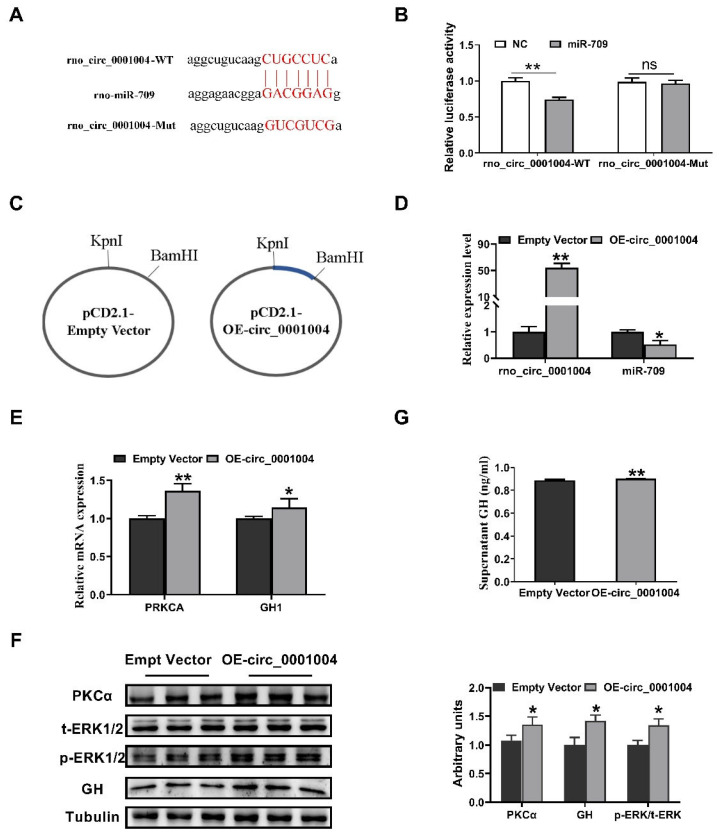
Rno_circ_0001004 antagonizes miR-709-mediated repression the synthesis and secretion of GH. (**A**) Bioinformatics target prediction. (**B**) Dual luciferase reporter gene assay verified. (**C**) Construction of rno_circ_0001004 overexpression vector. (**D**) Expression of rno_circ_0001004 along with miR-709 in empty vector and overexpression vector in GH_3_ by qRT-PCR. (**E**) mRNA level of PKCA and GH1 after transfection of OE-circ_0001004. (**F**) Western blot evaluation results of PKCα, t-ERK1/2, *p*-ERK1/2 and GH protein expression following transfection with rno_circ_0001004 in GH_3_ cells. (**G**) changes in supernatant GH level: average expression rose from 0.87 to 0.9 ng/mL. (* *p* < 0.05; ** *p* < 0.01).

Bioinformatics analysis with RNAhybird and miRanda was performed and indicated that rno_circ_0001004 has miR-709 binding sites (Figure 2A). Then, a dual-luciferase reporter assay showed that miR-709 overexpression was able to down-regulate the luciferase activity compared to miR-NC, and this inhibition was eliminated when the rno_circ_0001004 binding site was mutated (Figure 2B). To confirm their target relationship, the rno_circ_0001004 overexpression vector was constructed (Figure 2C), and after transfection into a GH_3_ cell, rno_circ_0001004 was overexpressed and miR-709 was correspondingly down-regulated (Figure 2D). The results above suggest that rno_circ_0001004 is a molecular sponge for miR-709. Our previous studies demonstrated that miR-709 significantly inhibited the GH synthesis by targeting PKCA [12]. Thus, we further detected the change in PKCα pathway and GH expression and release. Interestingly, our results show that rno_circ_0001004 obviously increases the mRNA and protein levels of PKCα, the phosphorylation of ERK1/2 (Figure 2E,F) and GH protein within cell as well as supernatant, compared to empty vectors (Figure 2F,G). These results confirm that rno_circ_0001004 antagonizes miR-709-mediated repression of the GH synthesis and secretion through the PKCα pathway.

### 2.3. Knockdown of Rno_circ_0001004 Suppresses the GH Synthesis and Secretion in GH_3_ Cell

**Figure 3:**
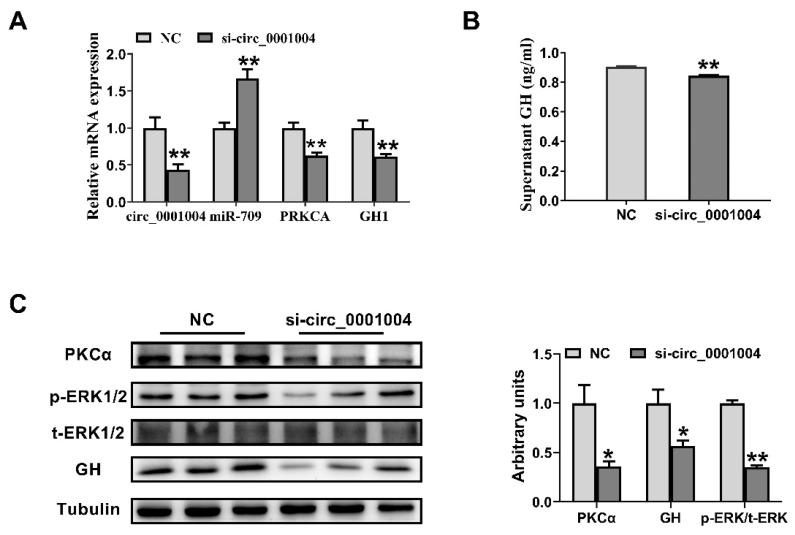
Knockdown of rno_circ_0001004 promoted the GH synthesis and suppressed GH_3_ cells. (**A**) The mRNA level of PKCA and GH1 along with circ_0001004 and miR-709 expression after transfection si-circ_0001004. (**B**) The changes in supernatant GH level after transfection si-circ_0001004. (**C**) The PKCα, t-ERK1/2, *p*-ERK1/2 and GH protein expression levels in GH_3_ cells after transfection si-circ_0001004. (* *p* < 0.05; ** *p* < 0.01).

In order to further verify the effect of rno_circ_0001004 on GH, we transfected si-circ_0001004 to GH_3_ cells. As expected, si-circ_0001004 significantly decreased the expression of circ_0001004, while it correspondingly increased the miR-709 expression level, followed by the inhibition of mRNA in *PRKCA* and *GH1* (Figure 3A). Furthermore, a Western blot revealed that the protein expression of PKCα, the phosphorylation of ERK1/2, and GH both in cell and supernatant all decreased with the inhibition of circ_0001004 (Figure 3B,C). The above results further confirm that rno_circ_0001004 regulates the synthesis and secretion of GH by acting as a molecular sponge for miR-709.

### 2.4. Rno_circ_0001004 Promoted the Viability of GH_3_ Cells

**Figure 4:**
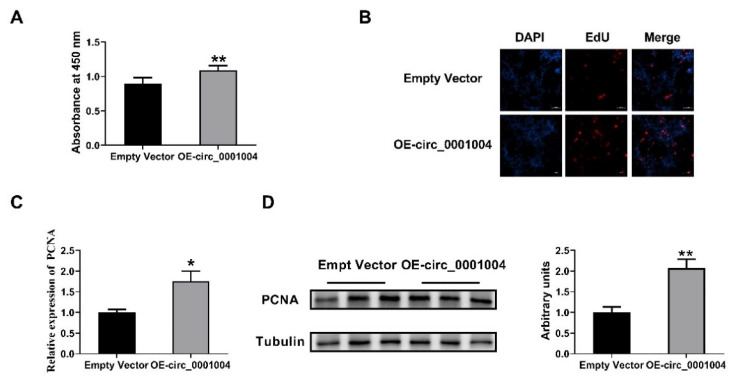
Rno_circ_0001004 promoted the viability of GH_3_ cells (**A**) The GH_3_ proliferation was evaluated with CCK-8 kits. (**B**) Cell proliferation tested by an EdU assay. (**C**) PCNA mRNA expression quantified by qRT-PCR. (**D**) Western blot assay for PCNA protein expression. (* *p* < 0.05; ** *p* < 0.01).

Our previous study showed that miR-709 suppressed the viability of GH_3_ cells [12]. Thus, we performed CCK8 and EdU assays to determine the effect of rno_circ_0001004 on GH_3_ cell proliferation. Intriguingly, circ_0001004 significantly promoted GH_3_ cell proliferation (Figure 4A,B). Moreover, PCNA, the key marker of cell proliferation, was markedly up-regulated both in mRNA and protein levels (Figure 4C,D).

### 2.5. Rno_circ_0001004 Reversed the Inhibition of Cell Proliferation by miR-709

**Figure 5:**
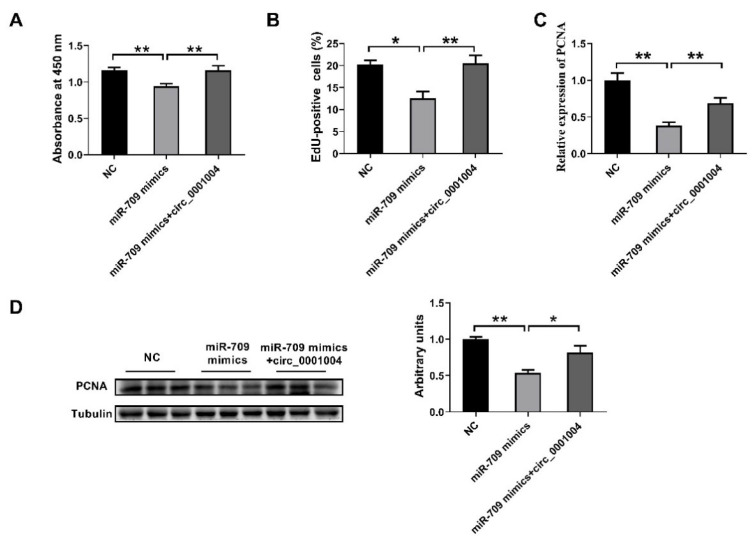
Effects of rno_circ_0001004 in reversing the inhibition of cell proliferation by miR-709. (**A**) The GH_3_ proliferation was evaluated with CCK-8 kits. (**B**) EdU-positive cells. (**C**) PCNA mRNA expression quantified by qRT-PCR. (**D**) Western blot assay for PCNA protein expression. (* *p* < 0.05; ** *p* < 0.01).

In order to further confirm the idea that rno_circ_0001004 serves as a ceRNA for miR709 to regulate the viability of GH_3_ cells, we next co-transfected miR-709 mimics and circ_0001004 into GH_3_ cells. The results demonstrates that overexpression of miR-709 obviously inhibits the cell proliferation of GH_3_, and this inhibition is perfectly rescued by overexpression of rno_circ_0001004, as shown by CCK8 assay (Figure 5A), EdU assay (Figure 5B) and PCNA expression (Figure 5C,D). These results provide more profound evidence that rno_circ_0001004 is a sponge ceRNA for miR-709.

## 3. Discussion

The pituitary gland, called the ‘master gland’ of the endocrine system, is the central regulator for growth, reproduction, metabolism and stress response [35]. The anterior pituitary accounts for 80% of the entire pituitary gland and secretes six major hormones, which are crucial to our physiological well-being [36,37]. As a major hormone in the pituitary gland, GH plays an important role in regulating the growth and metabolism of organisms [38,39]. 

MiRNAs are a class of small ncRNA with a length of about 22 nucleotides that post-transcriptionally regulate gene expression [40]. MiRNA generally functions primarily by binding to the 3′ untranslated region (UTR) of the target mRNA [41]. They play important roles in essential processes such as cell proliferation, cell apoptosis and cell differentiation [42,43]. miRNA was reported to participate in regulating GH. MiR-34b, miR-326, miR-432, miR-548c and miR-570 were found to regulate pituitary cell proliferation [44]. miR-126 played an important role in the development of GH-secreting pituitary adenomas [2]. Our previous study showed that miR-709 inhibited the GH synthesis and suppressed the viability of GH_3_ cells by targeting PRKCA [12]. 

CircRNAs are derived from the exon or intron region of genes [45]. There are currently three hypothetical models for the mechanism of circRNA formation, including lariat-driven circularization, intron pairing-driven circularization, and RNA binding protein (RBP)-mediated circularization [46]. CircRNAs containing multiple competing miRNA binding sites are likely to act as ceRNA in reducing miRNA activity and up-regulating the expression of miRNA-related target genes [47]. Thousands of strongly and stably expressed circRNAs have been detected [26]. CircFGFR4 promotes the differentiation of myoblasts by binding miR-107 [48]. Circ0005276 can promote the proliferation and migration of prostate cancer cells [49]. Circ-ZNF609 regulates nasopharyngeal carcinoma cell growth via modulating miR-188 expression [50]. Some studies have identified circRNAs in the pituitary gland [51,52,53,54]. At present, there have been few reports of circRNA regulating GH.

In the present study, we firstly noted that rno_circ_0001004 has the potential to act as miR-709 binding sites. Then, characterization of rno_circ_0001004 was carried out and the target relationship with miR-709 was validated using a dual luciferase assay. To further probe the influence of rno_circ_0001004 on GH, the overexpression of a rno_circ_0001004 vector and si-circ_0001004 were constructed. Next, the genes and proteins in the pathway of miR-709 regulation of GH were all evaluated. The results show that the expression trend of rno_circ_0001004 is almost opposite to that of miR-709, but is consistent with the expression trend of PKCα, GH and the phosphorylation of ERK1/2. Thus, our findings firstly clarify the molecular mechanism by which rno_circ_001004 can act as a sponge for miR-709 in regulating the synthesis and secretion of GH, providing novel insight into the regulatory mechanism of GH.

Our prior study also found that miR-709 repressed the viability of GH_3_ cells [12]. Thus, we explored whether rno_circR_0001004 affected the proliferation of GH_3_ cells. Compared with the empty vector, the rno_circ_0001004 overexpression group can significantly promote the proliferation of GH_3_ cells, as determined by the CCK-8 assay, PCNA expression and the EdU incorporation assay. Moreover, rno_circ_0001004 was able to perfectly reverse the inhibitory effect of miR-709 on the proliferation of GH_3_ cells. Therefore, our study reveals that rno_circ_001004 is able to positively regulate GH_3_ cell viability. These results indicate that rno_circ_0001004 plays an essential role in the regulation GH and pituitary cell proliferation. Furthermore, they lay a foundation for further study to explore the importance of circ_0001004 in regulation of animal and human growth and development.

## 4. Materials and Methods

### 4.1. Cell Culture and Transfection

GH_3_ cell line (ATCC) was cultured in F12 (Gibco, New York, NY, USA) medium supplemented with 2.5% fetal bovine serum (FBS) (Gibco, New York, NY, USA), 15% horse serum (Hyclone, Logan, UT, USA) and 1% penicillin/streptomycin (Gibco, New York, NY, USA). Hela cells were cultured in PRMI 1640 (Gibco, New York, NY, USA) culture medium with 10% FBS and 1% penicillin/streptomycin. GH_3_ cells were transfected with miR-709 mimic, rno_circ_0001004 or si-circ0001004 using Lipofectamine 2000 (Invitrogen, Carlsbad, CA, USA). The cells were incubated at 37 °C in a humidified atmosphere of 5% CO_2_.

### 4.2. RAN Isolation, cDNA Synthesis, RT-PCR and Sanger Sequencing

Total RNA was isolated from GH_3_ cells by Trizol reagent (Invitrogen, Carlsbad, CA, USA) according to the manufacturer’s protocol. The cDNAs were obtained by Color Reverse Transcription Kit (with gDNA remover) (EZBioscience, Roseville, CA, USA). Genomic DNA (gDNA) was extracted using a Genomic DNA Isolation Kit (Sangon Biotech, Shanghai, China). Quantification of mRNA, miRNA, circRNA and gDNA was performed by using a SBRY Green PCR Kit (Takara, Tokyo, Japan), primers and Real-Time PCR System (Bio-Rad Laboratories, Inc., Hercules, CA, USA) The circRNA and mRNA levels were normalized to those of β-actin, while the miR-709 levels were normalized to the U6 and determined by 2-DDCt method. The primer sequences for the amplification of specific primers are listed in supplementary Table S1. Sanger sequencing (chain termination sequencing) is a method of DNA sequencing based upon the selective incorporation of chain-terminating dideoxynucleotides (ddNTPs) during in vitro DNA replication [55].

### 4.3. Vector Construction

The sequence for exons 9–11 of Wnk2 was PCR amplified using primers F (5′-GGGGTACCTGAAATATGCTATCTTACAGCCTGGCCTATCAGTGGGC-3′) and R (5′-CGGGATCCTCAAGAAAAAATATATTCACCTGGGTCCCTGAGGCAGC-3′), then cloned into KpnI and BamHI restriction sites of a circular expression vector, the pcd2.1-ciR (GENESEED, Guangzhou, China), by digestion to create rno_circ_0001004-overespressing vector.

### 4.4. Dual-Luciferase Reporter Assay

Hela cells were seeded in 96-well cell culture plates. When their confluence reached about 80%, the miR-709 mimic and rno_circ_0001004-Wt or rno_circ_0001004-Mut were co-transfected into cells using Lipofectamine 2000. After incubation for 48 h, the cells were washed with PBS and the luciferase activity was measured by the Dual-GLO luciferase reporter assay system (Promega, Madison, WI, USA) according to the manufacturer’s instructions. 

### 4.5. Evaluation of GH_3_ Proliferation

GH_3_ proliferation was assessed by the cell counting kit-8 (CCK-8) method, 5-ethynyl-2′ deoxy uridine (EdU) incorporation assay and proliferating cell nuclear antigen (PCNA) expression. Firstly, the rate of GH_3_ proliferation was determined with the CCK-8 kit (Bioss, Beijing, China) according to the manufacturer’s instructions. The number of viable cells was assessed by measuring the absorbance at 450 nm using a Synergy 2 Multi-Mode Reader (Bio Tek Instruments, Inc., Winooski, VT, USA). Secondly, DNA synthesis was examined with EdU incorporation assay (YF^®^ 555 Click-iT EdU Imaging Kit, Suzhou US EVERBRIGHT, Suzhou, China) to evaluate GH_3_ proliferation. The EdU positive cells were counted and normalized by the total number of Hoechst 33,342 stained cells. Lastly, GH_3_ proliferation was evaluated by PCNA expression, which is the auxiliary component of DNA polymerase δ and constitutes a useful proliferation marker.

### 4.6. Western Blot Analysis

GH_3_ cells were lysed in a RIPA lysis buffer (Beyotime Institute of Biotechnology, Shanghai, China) containing 1mM phenyl methane sulfonyl fluoride (PMSF). The concentration of protein was measured using the BCA Protein Assay Kit (Thermo Fisher Scientific, Waltham, MA, USA) according to the manufacturer’s instructions. Equal amounts of total protein were separated by SDS-PAGE and transferred to a PVDF membrane in a tris-glycine methanol buffer. The primary antibodies used in this study were as follows: GH monoclonal antibody (sc-374266, Santa Cruz, CA, USA), PKCα polyclonal antibody (BS1577, Bioworld, St. Louis Park, MN, USA), ERK1/2 monoclonal antibody (4695, CST, Danvers, MA, USA), Phospho-ERK1/2 monoclonal antibody (Tyr204) (4370, CST, Danvers, MA, USA), PCNA monoclonal antibody (200947-2E1, ZEN BIO, Chengdu, China) and Tubulin polyclonal antibody (AP0064, Bioworld, St. Louis Park, MN, USA), HRP conjugated goat anti-rabbit IgG (BS13278, Bioworld, St. Louis Park, MN, USA) and HRP conjugated goat anti-mouse IgG (BS12478, Bioworld, St. Louis Park, MN, USA) were used as secondary antibodies. The membranes were incubated with ImmobilonTM Western Chemiluminescent HPR Substrate (Millipore, Burlington, WA, USA) and scanned with a FlourChem M Fluorescent Western Imaging System (Protein Simple, Santa Clara, CA, USA). The protein band density was determined by the software Image J and normalized with a corresponding Tubulin intensity.

### 4.7. Quantification of Secretory GH by ELISA

The concentration of GH in a cell medium of GH_3_ cells transfected with rno_circ_0001004 and si-circ_0001004 was determined using the reagents in the Rat Growth Hormone ELISA kit (Enzyme-linked Biotechnology, Shanghai, China) according to the manufacturer’s protocols. Color alterations in the wells were read using the 96-well microplate reader (Bio Tek Instruments, Inc., Winooski, VT, USA).

### 4.8. Statistics Analysis

All experimental results are presented as the mean ± S.E.M, with at least three independent replications. Statistical analysis was performed using SPSS 17.0 software. The statistically significant differences among groups were tested by one-way analysis of variance (ANOVA). *p* < 0.05 was considered as statistically significant. * *p* < 0.05; ** *p* < 0.01.

## 5. Conclusions

**Figure 6:**
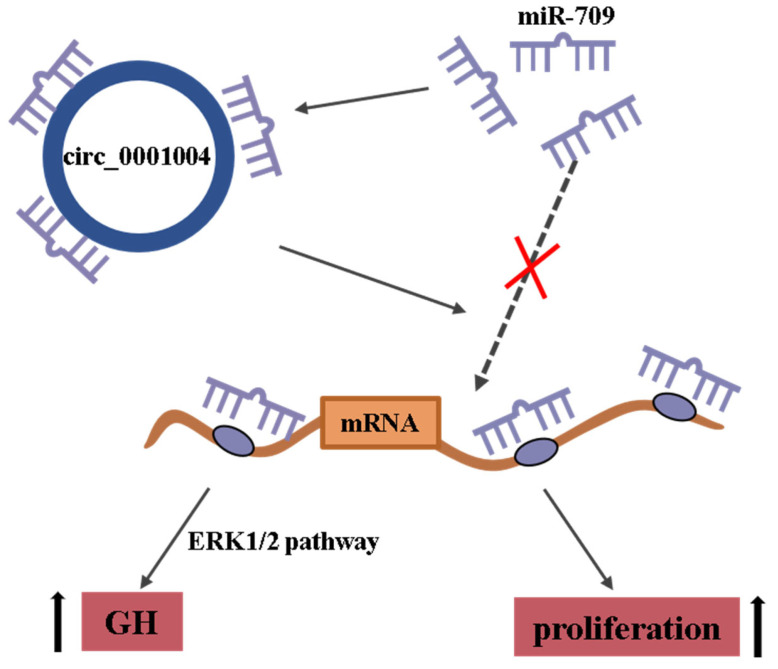
Rno_circ_0001004 has 3 miR-709 binding sites and can act as a miR-709 molecular sponge to regulate GH synthesis and cell proliferation.

In summary, our study reveals that rno_circ_0001004 competitively binds miR-709 to regulate the GH synthesis and cell proliferation in rat pituitary cells (Figure 6). To the best of our knowledge, our findings are the first case to illustrate regulation of GH by circRNA_0001004 and provide novel evidence on the circRNA-miRNA-mRNA network in pituitary cells.

## Data Availability

Not applicable.

## Supplementary Materials

The following supporting information can be downloaded at: https://www.mdpi.com/article/10.3390/ijms23031413/s1.
