# Supplementary material for: Rno_circ_0001004 Acts as a miR-709 Molecular Sponge to Regulate the Growth Hormone Synthesis and Cell Proliferation
Source: Int J Mol Sci. 2022 Jan 26;23(3):1413. doi: 10.3390/ijms23031413 (PMC8835962; doi:10.3390/ijms23031413)
Supplement: Supplementary file 1 [file ijms-23-01413-s001.zip › ijms-1552949-supplementary.pdf]

Supplementary Table S1 Primers and sequences used in this study

Primers		
GH1	F	TCAGCAGGATCTTTACCAACAG
	R	GCAGGAGAGCAGCCCATAG
PRKCA	F	AGGGACCTGACACTGATGACC
	R	TTCATCTGTGACCTCTGCCTTC
β-actin	F	TGTGCCCATCTATGAGGGTTAC
	R	ACGGATGTCAACGTCACACTTC
miR-709	F	GGAGGCAGAGGCAAGAGG
	R	ATCCAGTGCGTGTCGTGGA
U6	F	TGCTTCGGCAGCACATATAC
	R	TTCACGAATTTGCGTGTCAT
miR-709 mimics	F	GGAGGCAGAGGCAAGAGGA
	R	CUCUUGCCUCUGCCUCCUU
NC mimic	F	UUCUCCGAACGUGUCACGUTT
	R	ACGUGACACGUUCGGAGAATT
Circ_0001004	F	CCTCGTCTGTGCCTCCTCA
	R	GAAGGCTCTGGAAAATGCTG
Si-circ_0001004	F	CCCAGCCUGGCCUAUCAGUTT
	R	ACUGAUAGGCCAGGCUGGGTT
